# Multidisciplinary Management of a Giant Ovarian Sertoli–Leydig Cell Tumor Presenting With Isosexual Precocious Puberty: A Pediatric Case Report

**DOI:** 10.1002/ccr3.73700

**Published:** 2026-10-15

**Authors:** Sanaa Bouramdane, Kenza Elmkaddem, Nawal Hammas, Meryem Boubbou, Othmane Alaoui, Khalid Khattala, Youssef Bouabdellah, Sarra Benmiloud

**Affiliations:** ^1^ Department of Pediatric Hematology and Oncology, Hassan II University Hospital, Faculty of Medicine and Pharmacy Sidi Mohamed Ben Abdellah University Fez Morocco; ^2^ Department of Pathology, Hassan II University Hospital, Faculty of Medicine and Pharmacy Sidi Mohamed Ben Abdellah University Fez Morocco; ^3^ Department of Mother and Child Radiology, Hassan II University Hospital, Faculty of Medicine and Pharmacy Sidi Mohamed Ben Abdellah University Fez Morocco; ^4^ Department of Pediatric Surgery, Hassan II University Hospital, Faculty of Medicine and Pharmacy Sidi Mohamed Ben Abdellah University Fez Morocco

**Keywords:** inhibin B, neoadjuvant chemotherapy, pediatric ovarian tumor, precocious puberty, Sertoli–Leydig cell tumor, surgery

## Abstract

Sertoli–Leydig cell tumors (SLCTs) are rare ovarian sex cord‐stromal tumors, particularly uncommon in the pediatric population. They are typically androgen‐secreting, while estrogen‐secreting forms are rare and may lead to diagnostic confusion with granulosa cell tumors. We report the case of a 7‐year‐old girl presenting with progressive abdominal distension and isosexual precocious puberty. Imaging revealed a large abdominopelvic mass of uncertain origin. Histopathological examination of a biopsy suggested a sex cord‐stromal tumor. The patient received neoadjuvant chemotherapy with the VIP regimen, resulting in significant tumor reduction, followed by fertility‐sparing surgery. Final histopathological and immunohistochemical analysis confirmed a SLCT. The tumor was classified as FIGO stage IC2 due to preoperative capsular breach. Postoperatively, an additional cycle of chemotherapy was administered. Hormonal markers, including estradiol and inhibin B, normalized after treatment. During follow‐up, a transient rise in these markers was observed in the absence of radiological abnormalities, followed by spontaneous normalization. The patient remains in complete remission after 27 months of follow‐up. This case highlights an atypical estrogen‐secreting pediatric SLCT and underscores the importance of histopathological confirmation in unusual clinical presentations. It also illustrates the potential role of neoadjuvant chemotherapy in large tumors and supports the use of inhibin B as a biomarker for disease monitoring, although its interpretation should be cautious and integrated with clinical and imaging findings. Long‐term follow‐up is essential due to the risk of recurrence.

## Key Clinical Message

Pediatric estrogen‐secreting Sertoli–Leydig cell tumors may clinically mimic juvenile granulosa cell tumors. Histopathological confirmation remains essential for diagnosis. In selected cases, neoadjuvant chemotherapy may facilitate fertility‐sparing surgery, while transient elevations of hormonal markers should be interpreted cautiously and correlated with clinical and radiological findings during follow‐up.

## 1 Introduction

Sertoli–Leydig cell tumors (SLCTs) are rare ovarian sex cord‐stromal tumors (SCSTs), accounting for < 0.5% of all ovarian neoplasms. They occur predominantly in young women and represent approximately 4% of ovarian tumors occurring during childhood [1]. These tumors are frequently hormonally active and typically present with signs of hyperandrogenism or virilization [2]. However, in rare cases, they may secrete estrogens, leading to isosexual precocious puberty, particularly in pediatric patients [3]. The diagnosis of SLCTs relies on histopathological and immunohistochemical examination, as clinical and radiological findings are often non‐specific [4]. Recent studies have also highlighted an association with DICER1 gene mutations, suggesting a potential tumor predisposition syndrome [5]. We report a rare case of pediatric SLCT revealed by isosexual precocious puberty, managed with neoadjuvant chemotherapy followed by fertility‐sparing surgery, and we highlight the role of hormonal markers in disease monitoring.

## 2 Case History/Examination

We report the case of a 7‐year‐and‐4‐month‐old girl with no significant past medical history, who was initially evaluated at another healthcare facility after her family noticed progressive abdominal distension evolving over a 2‐month period. Intermittent vaginal bleeding was also reported during this period. Ultrasonography and contrast‐enhanced computed tomography (CT) of the abdomen and pelvis revealed a large, well‐defined, multiloculated solid‐cystic abdominopelvic mass with regular contours, measuring 135 × 85 × 165 mm and located in the prerectal region. The mass was in close contact with several adjacent structures, displacing the bladder anteriorly and the uterus laterally, with loss of the intervening fat planes with the bladder, abdominal wall, rectum, and adjacent bowel loops. The solid component was vascularized, and a small amount of free ascites was present. The exact organ of origin could not be determined. The imaging features initially raised suspicion of a sarcomatous lesion, and as the origin of the mass could not be determined, a biopsy was performed. Histopathological examination of the biopsy specimen revealed an undifferentiated neoplastic proliferation. Immunohistochemical staining showed positivity for calretinin and inhibin, suggestive of a SCST. The patient was subsequently referred to our institution for further management.

On physical examination, her height was at the 95th percentile and her weight at the 75th percentile. Abdominal examination revealed a large, firm, non‐tender, fixed left‐sided abdominopelvic mass measuring approximately 230 × 140 mm. Pubertal assessment showed Tanner Stage II breast development, Tanner Stage III pubic hair, and Tanner Stage I axillary hair, associated with intermittent vaginal bleeding. No signs of virilization or disorders of sexual differentiation were observed.

## 3 Methods

Magnetic resonance imaging (MRI) of the abdomen and pelvis with contrast revealed a large, well‐defined, lobulated abdominopelvic mass measuring 135 × 90 × 195 mm, with heterogeneous signal intensity on T2‐weighted images and both solid and cystic components (Figure 1). There was marked enlargement of the left ovarian vascular pedicle extending superiorly up to the ipsilateral renal vein and inferiorly toward the mass, suggesting a left ovarian origin. A small amount of intraperitoneal fluid was noted, without clearly identifiable peritoneal implants. The right ovary appeared normal, with no evidence of lymphadenopathy or other abnormalities in the upper abdomen. Chest CT revealed five pulmonary micronodules ranging from 2 to 5 mm in diameter, which were considered non‐specific for metastatic disease. Bone age was assessed using a radiograph of the left hand and wrist according to the Greulich and Pyle method and was consistent with the patient's chronological age.

**FIGURE 1:**
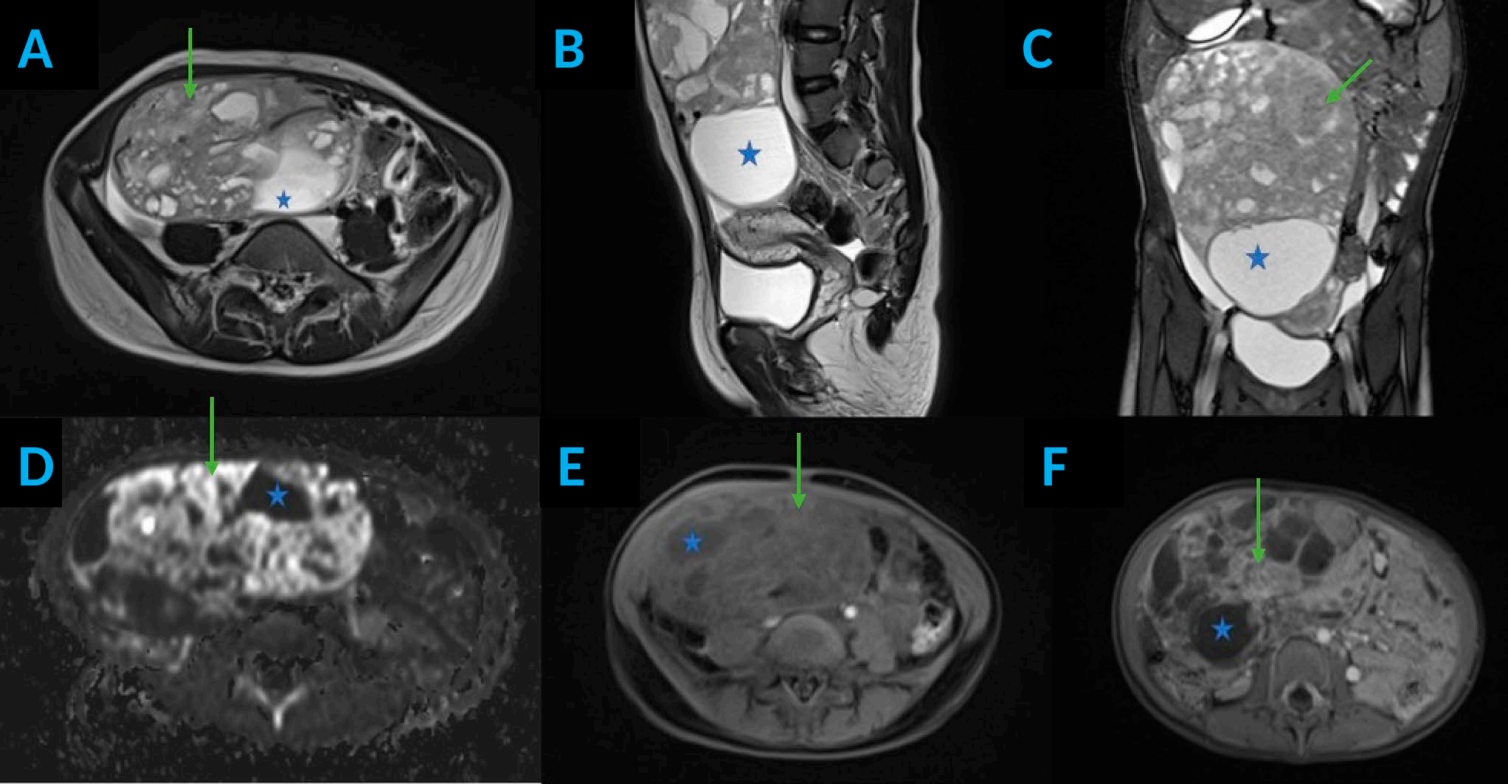
Pelvic MRI: Axial (A), sagittal (B), and coronal (C) T2‐weighted sequences, diffusion‐weighted imaging (D), pre‐contrast T1‐weighted imaging (E), and post‐contrast T1‐weighted imaging (F). A large midline supracervical pelvic mass with mixed solid and cystic components is demonstrated. The solid component (green arrow) shows intermediate T2 signal intensity, restricted diffusion, and heterogeneous contrast enhancement, whereas the cystic component (blue star) appears hyperintense on T2‐weighted images, hypointense on T1‐weighted images, and shows no diffusion restriction.

Given the associated pubertal signs, a hormonal evaluation was performed and revealed markedly elevated levels of estradiol, inhibin B, and anti‐Müllerian hormone (AMH), with suppressed gonadotropins, consistent with a peripheral (gonadotropin‐independent) cause of precocious puberty. A moderate elevation of testosterone and delta‐4‐androstenedione levels was observed (Table 1). Serum alpha‐fetoprotein (AFP) and beta‐human chorionic gonadotropin (β‐hCG) levels were within normal limits. Taken together, the clinical findings and hormonal profile were consistent with peripheral (gonadotropin‐independent) isosexual precocious puberty, supporting a hormone‐secreting ovarian tumor.

**TABLE 1:** Hormonal profile at diagnosis and during treatment.

Parameter	Unit	Normal range	At diagnosis	After 3 VIP cycles	Postoperative	End of treatment
AMH	ng/mL	1.88–7.29	30.2	3.05	0.05	< 0.01
FSH	mIU/mL	< 0.3	< 0.30	—	—	5.73
LH	mIU/mL	< 1.5	< 0.1	—	—	0.74
Testosterone	ng/mL	< 0.3	0.479	< 0.025	< 0.025	< 0.025
Androstenedione (Δ4)	ng/mL	0.150–0.519	0.862	0.175	0.2	0.162
DHEA‐S	μg/dL	2.8–85.2	51.65	—	—	—
Estradiol	pg/mL	< 35	378	12.79	20.86	6.64
Inhibin B	pg/mL	30–340	> 5000	580	< 15	< 15

Abbreviations: AMH, anti‐Müllerian hormone; DHEA‐S, dehydroepiandrosterone sulfate; FSH, follicle‐stimulating hormone; LH, luteinizing hormone; VIP, etoposide, ifosfamide, and cisplatin regimen.

Based on the biopsy findings, imaging features, and hormonal profile, the working diagnosis was a malignant hormone‐secreting ovarian SCST. Given the very large tumor size and its close relationship with adjacent pelvic structures, upfront complete resection was considered challenging. Following multidisciplinary discussion, neoadjuvant chemotherapy was therefore selected to achieve tumor shrinkage and facilitate subsequent complete fertility‐sparing surgery. The patient received three cycles of the VIP (etoposide, ifosfamide, and cisplatin) regimen (etoposide 75 mg/m^2^ on Days 1–5, ifosfamide 3000 mg/m^2^ on Days 1–2, and cisplatin 20 mg/m^2^ on Days 1–5) every 21 days. No specific fertility‐preservation procedure was performed before initiation of chemotherapy. Follow‐up contrast‐enhanced CT demonstrated approximately 65% tumor reduction, accompanied by a significant decrease in hormonal levels (Table 1).

The patient subsequently underwent laparotomy. Surgical exploration revealed a large encapsulated mass arising from the left ovary and adherent to the omentum (Figure 2). The uterus, contralateral ovary, and peritoneum appeared macroscopically normal. Peritoneal fluid was collected for cytological examination. A left salpingo‐oophorectomy was performed, and the adherent omental segment was resected for histopathological assessment.

**FIGURE 2:**
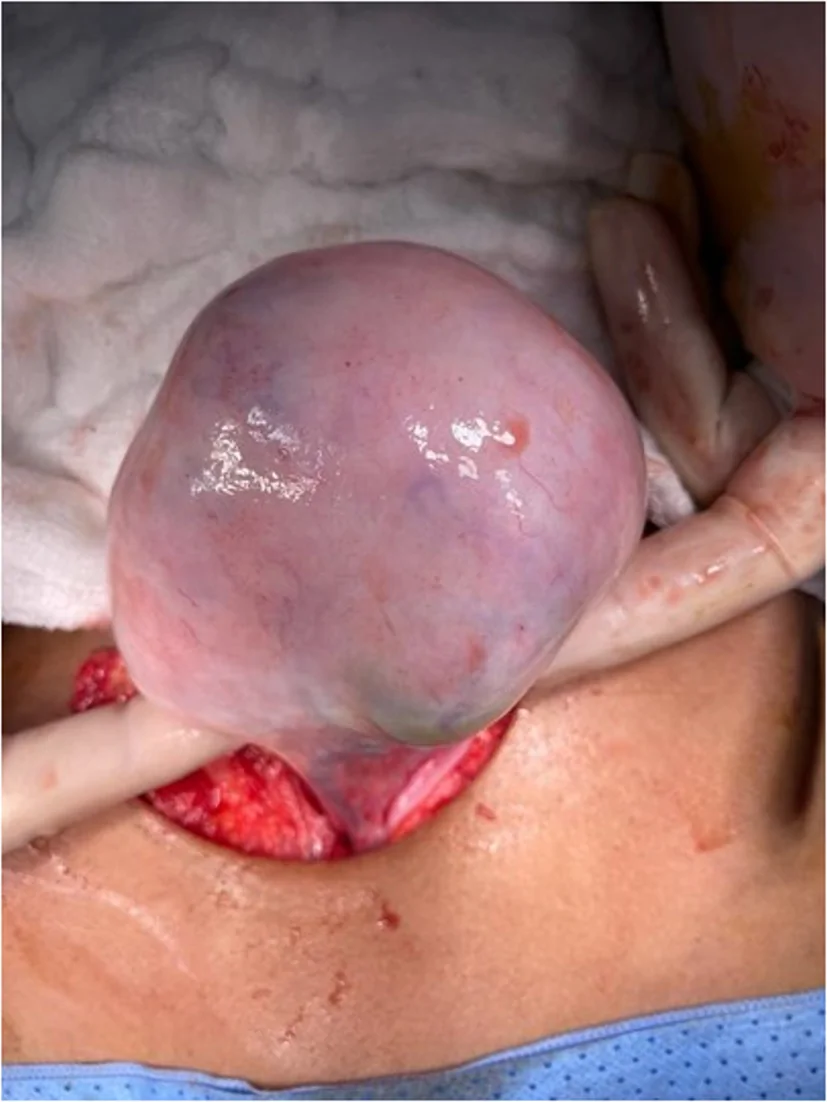
Intraoperative findings. Large encapsulated tumor arising from the left ovary.

On gross examination, the tumor measured 100 × 90 mm. On sectioning, it showed a solid‐cystic appearance with a homogeneous beige cut surface. Histopathological examination demonstrated a tumor composed of solid sheets and nests with cystic and microcystic areas (Figure 3). Tumor cells were round to polygonal, with moderately enlarged nuclei, fine chromatin, and clear cytoplasm (Figure 4). Mitotic activity was increased. A therapeutic response of approximately 70% was observed, characterized by fibro‐inflammatory changes, necrosis, and calcifications. The initial histological impression was that of a SCST suggestive of a juvenile granulosa cell tumor. However, immunohistochemical findings, including androgen receptor expression, supported the diagnosis of a residual SLCT (Figure 5). Histopathological examination of the resected omental tissue showed no pathological abnormalities and no evidence of tumor involvement. No extra‐ovarian extension was identified, and peritoneal cytology was negative for malignant cells.

**FIGURE 3:**
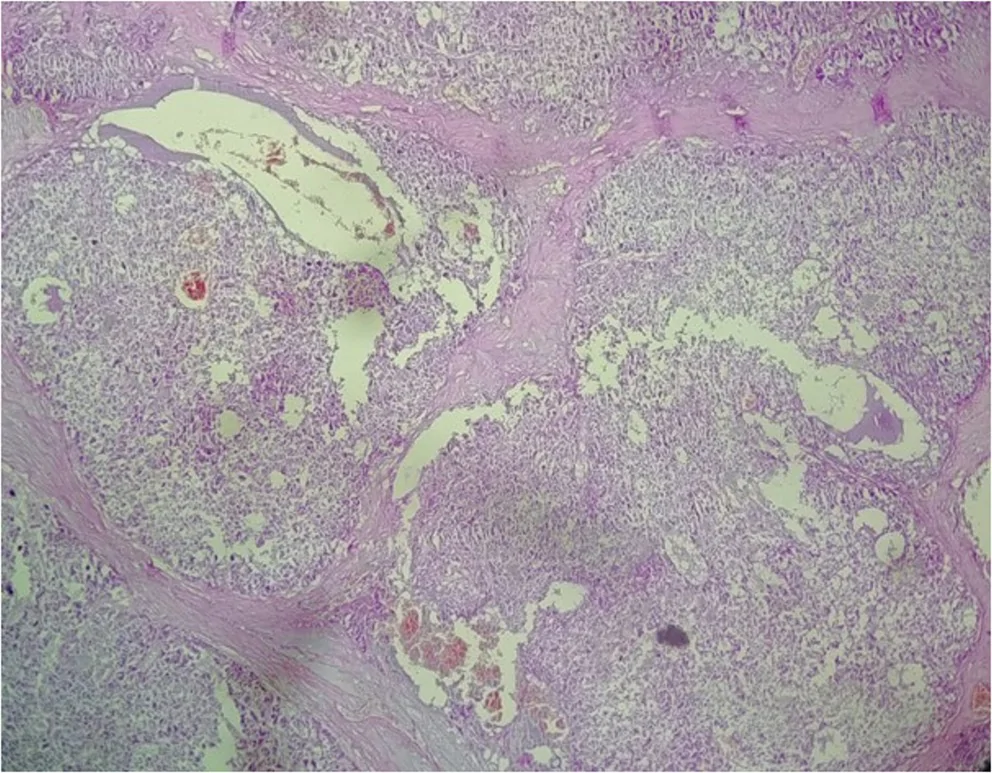
Tumor proliferation arranged in solid sheets (H&E, ×40).

**FIGURE 4:**
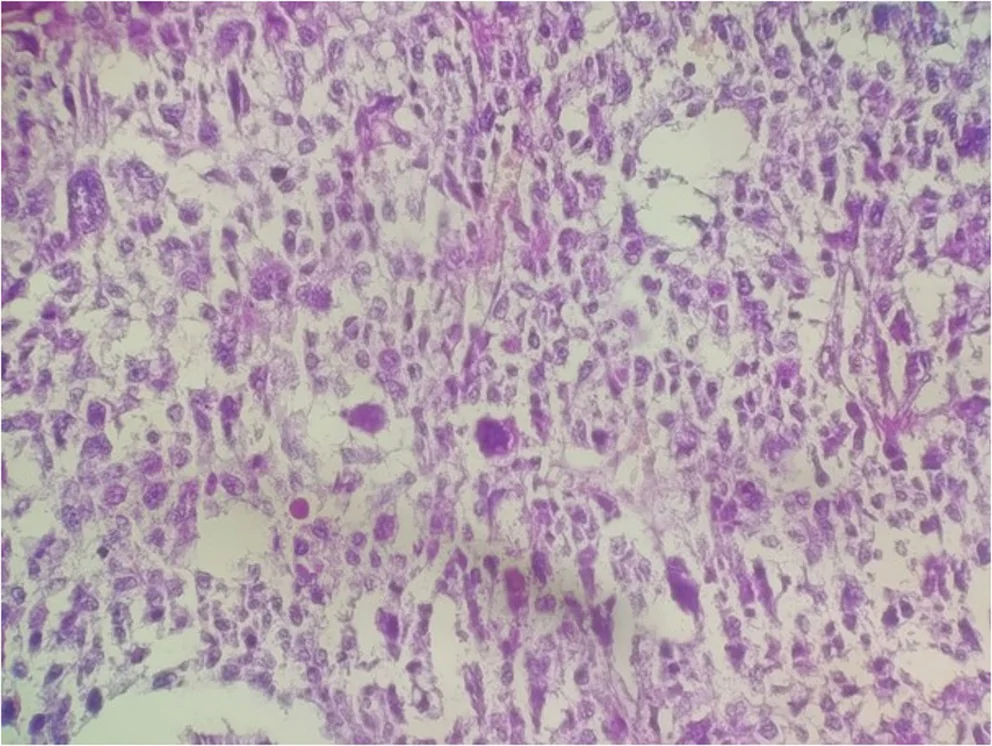
Round to polygonal tumor cells with eosinophilic or clear cytoplasm (H&E, ×200).

**FIGURE 5:**
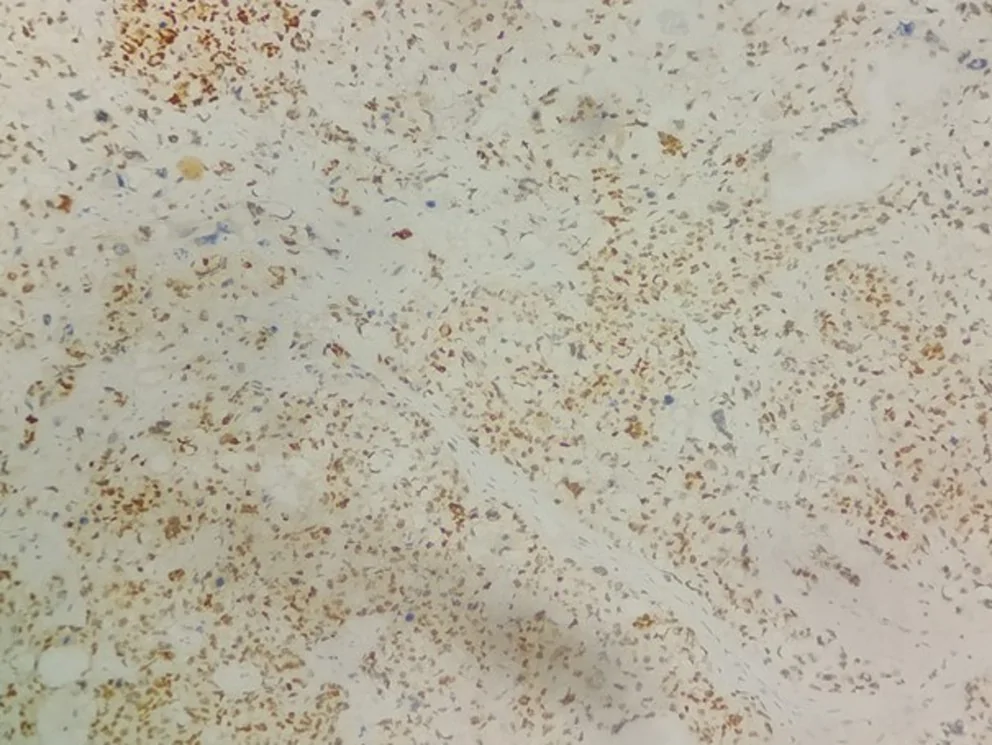
Immunohistochemical analysis demonstrating positive androgen receptor expression in tumor cells.

According to the International Federation of Gynecology and Obstetrics (FIGO) classification, the tumor was staged as stage IC2 due to preoperative capsular breach related to transabdominal biopsy.

In the postoperative setting, considering the confirmed diagnosis and the FIGO stage, a multidisciplinary decision was made to administer a fourth cycle of chemotherapy using the VIP regimen.

## 4 Conclusions and Results

End‐of‐treatment contrasted‐enhanced chest, abdominal, and pelvic CT showed no residual pelvic mass, lymphadenopathy, distant metastatic lesions, or ascites. Estradiol, inhibin B, testosterone, and delta‐4‐androstenedione levels had normalized, gonadotropins were no longer suppressed, and AMH had decreased to < 0.01 ng/mL (Table 1). The patient was subsequently placed on regular follow‐up every 3 months, including clinical assessment, imaging studies (abdominal ultrasound and chest radiography), and hormonal evaluation.

Fifteen months after completion of treatment, a rise in inhibin B and estradiol levels was observed as shown in Figures 6 and 7. Thoracoabdominal‐pelvic CT did not reveal any abnormalities, except for two pulmonary micronodules measuring 3 and 6 mm. A positron emission tomography (PET) scan showed no significant abnormalities. In the absence of radiological evidence of recurrence, a decision was made to continue close surveillance. A repeat hormonal assessment demonstrated normalization of all previously elevated markers (Figures 6 and 7).

**FIGURE 6:**
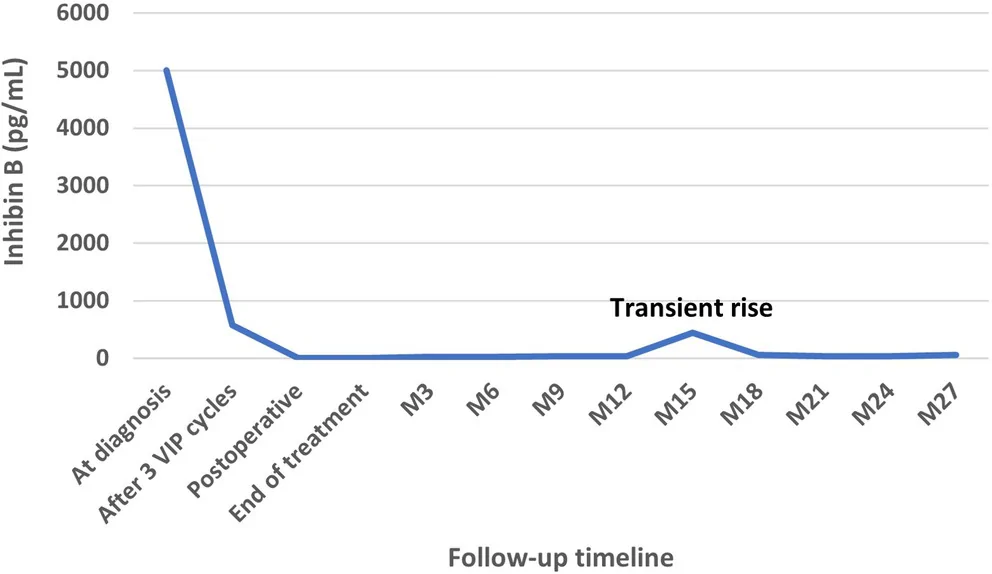
Kinetics of inhibin B during treatment and follow‐up.

**FIGURE 7:**
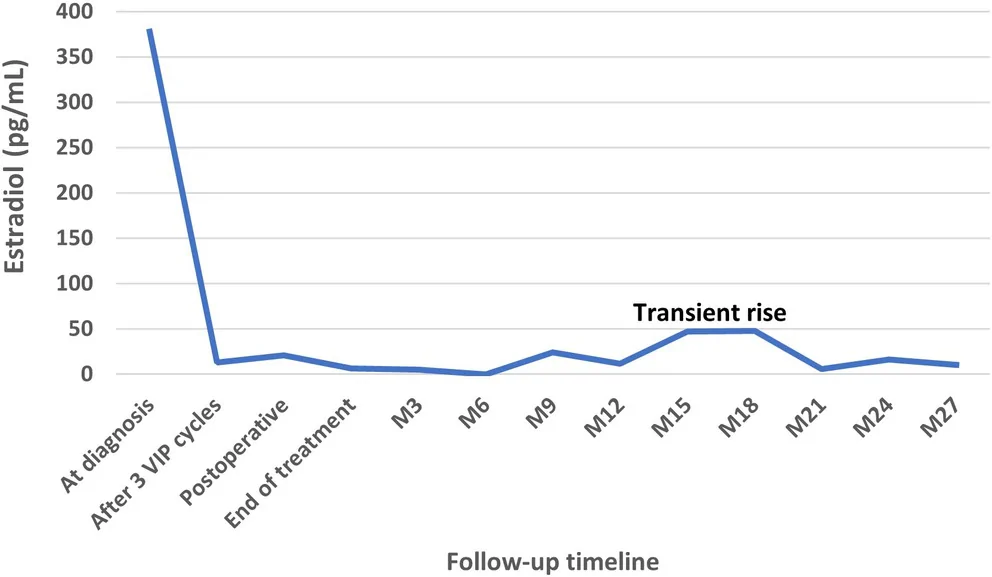
Kinetics of estradiol during treatment and follow‐up.

The patient is currently in complete clinical, biological, and radiological remission, with a follow‐up duration of 27 months. The premature breast development progressively regressed during follow‐up.

## 5 Discussion

Ovarian malignancies are rare in the pediatric population, accounting for approximately 1% of all childhood cancers. SCSTs, as a group, comprise approximately 7% of all ovarian tumors and nearly 15% of pediatric ovarian tumors [6]. This heterogeneous group includes granulosa cell tumors (GCTs), SLCTs, thecomas, fibromas, and other rare entities. In children, juvenile granulosa cell tumors (JGCTs) are the most frequent subtype, followed by SLCTs [7].

SLCTs are exceptionally rare, accounting for < 0.5% of ovarian tumors, and occur predominantly in adolescents and young adults [1, 8]. Their occurrence in young children, as in our case, is uncommon and places this observation within a particularly rare subgroup. Only a few cases of SLCTs have been reported in children under the age of 8 years in the literature so far [3]. This rarity contributes to the lack of standardized pediatric‐specific management and the reliance on data extrapolated from adult series [9].

Clinically, SLCTs are usually hormonally active. In approximately 40%–60% of cases, they present with androgenic manifestations, including hirsutism, acne, deepening of the voice, amenorrhea, clitoromegaly, or increased muscle mass [2, 9]. In contrast, estrogen‐secreting forms are uncommon and may present in children with isosexual precocious puberty, vaginal bleeding, or premature breast development [6, 10]. Our patient presented with a predominantly estrogenic phenotype, including breast development, pubic and axillary hair, and intermittent vaginal bleeding, without hirsutism or clear signs of virilization. This presentation is unusual for SLCT and clinically overlaps with JGCT, which contributed to the initial diagnostic uncertainty. This case therefore illustrates the diagnostic challenge of estrogen‐secreting pediatric SLCTs and highlights the essential role of histopathological and immunohistochemical confirmation.

Hormonal evaluation plays a crucial role in this context, both for diagnostic orientation and for distinguishing ovarian from adrenal steroid secretion. In classical SLCTs, elevated testosterone levels are frequently observed, whereas normal dehydroepiandrosterone sulfate (DHEA‐S) levels favor an ovarian rather than adrenal origin [2]. In our patient, there was a moderate elevation of testosterone and delta‐4‐androstenedione, with normal DHEA‐S levels, supporting an ovarian origin, consistent with the literature. However, the most striking feature was the marked elevation of estradiol associated with suppressed luteinizing hormone (LH) and follicle‐stimulating hormone (FSH), consistent with peripheral precocious puberty. This hormonal profile is much less common in SLCTs than in GCTs, highlighting the originality of our case. Additionally, our patient showed marked elevation of inhibin B and AMH at diagnosis, both of which decreased markedly following treatment, supporting their role as markers of tumor activity. Compared with the literature, our case is characterized by the coexistence of estrogen hypersecretion and markedly elevated inhibin B and AMH levels, providing a particularly illustrative biological profile with strong relevance for disease monitoring [11]. During follow‐up, a transient rise in inhibin B and estradiol was observed in the absence of radiological abnormalities, followed by spontaneous normalization. This observation suggests that inhibin B may be a sensitive marker of disease activity and may increase before radiological abnormalities become evident. However, isolated elevations should be interpreted cautiously and correlated with clinical and imaging findings.

Imaging plays a key role in the initial assessment. In pediatric patients, ultrasound is the first‐line modality, while MRI provides better tissue characterization and evaluation of local extension [12]. Features suggestive of malignancy include tumor size > 12 cm, hypervascularization, and the presence of ascites. In reported SLCT series, imaging typically shows a unilateral, well‐circumscribed, solid or mixed solid‐cystic mass without specific distinguishing features [2, 12]. In our case, MRI revealed a large abdominopelvic mass with heterogeneous solid and cystic components, without definite signs of local invasion associated with minimal ascites and no visible peritoneal implants. The presence of a prominent vascular pedicle arising from the left ovary was a key element suggesting an ovarian origin. While this is consistent with previously reported findings, the tumor measuring approximately 20 cm at diagnosis was exceptionally large compared to most published cases, although giant forms have been described in children and adolescents.

The definitive diagnosis of SLCT relies on histopathological examination. These tumors are composed of Sertoli cells arranged in cords or tubules and Leydig cells dispersed within a fibrous stroma. They are classified into well‐differentiated, moderately differentiated, poorly differentiated, retiform, and heterologous variants [7]. Immunohistochemically, positivity for inhibin and calretinin is characteristic and supports the diagnosis of a SCST [4, 13]. In our patient, histopathological examination confirmed the diagnosis of SLCT, supported by the expression of inhibin, calretinin, and androgen receptors. These findings were decisive in establishing the final diagnosis, particularly given the atypical estrogenic presentation that initially raised the possibility of a JGCT.

From a staging perspective, the FIGO classification remains the reference system for ovarian tumors. Stage I disease is confined to the ovary, with stage IC including tumors with capsular rupture, surface involvement, or malignant cells in ascites or peritoneal washings [14]. In our patient, the tumor was classified as FIGO stage IC2 due to preoperative capsular breach related to transabdominal biopsy. This classification has important prognostic and therapeutic implications, as capsular rupture is considered a risk factor for recurrence.

The mainstay of treatment for SLCTs is surgical resection [11]. In stage I disease, fertility‐sparing surgery with unilateral salpingo‐oophorectomy is recommended in young patients. However, the indication for adjuvant chemotherapy depends on several factors, including FIGO stage, tumor differentiation, tumor rupture, and histological aggressiveness [9, 11]. Chemotherapy is generally recommended in cases of stage IC or higher disease, poorly differentiated tumors, or the presence of heterologous elements [2, 9, 14]. In our case, the management strategy was individualized due to the very large tumor size and initial diagnostic uncertainty. Neoadjuvant chemotherapy using the VIP regimen was administered, resulting in significant tumor reduction and allowing complete surgical resection. Postoperatively, an additional cycle of chemotherapy was administered in view of the FIGO IC2 stage due to the presence of preoperative capsular breach as recommended [15]. Although there is no universally established standard chemotherapy regimen for SLCTs, platinum‐based combinations are most commonly used. The BEP regimen (bleomycin, etoposide, and cisplatin) is the most widely reported protocol, while VIP represents a valid alternative, particularly in cases where bleomycin is contraindicated or avoided [9, 14]. Both regimens have demonstrated efficacy in SCSTs, although evidence remains limited due to the rarity of these tumors [16].

Prognosis in SLCTs depends on several factors, including FIGO stage, degree of tumor differentiation, presence of capsular rupture, and histological features such as heterologous elements [14, 15, 16]. Most tumors diagnosed at stage I have a favorable outcome, with reported overall survival rates exceeding 90% in well‐differentiated forms [14, 15]. In contrast, advanced FIGO stage (≥ IC) and unfavorable histological features are associated with an increased risk of recurrence and poorer prognosis [14, 16]. In our case, the presence of capsular breach together with the undifferentiated histological features further supported the indication for adjuvant chemotherapy and close surveillance, despite the favorable clinical course [15, 16]. Recurrence rates range from 10% to 20%, with most relapses occurring within the first 3 years following initial treatment, supporting the need for close and prolonged follow‐up [14, 17]. Surveillance should include clinical evaluation, imaging, and hormonal monitoring [17]. In our patient, the transient hormonal elevation observed in the absence of radiological abnormalities raises the possibility of minimal residual disease or biological fluctuation. This finding highlights the complementary role of hormonal markers and imaging in follow‐up strategies.

Finally, recent studies have demonstrated an association between SLCTs and mutations in the DICER1 gene, particularly in pediatric cases [4, 5]. Pathogenic germline DICER1 variants are associated with a tumor predisposition syndrome that may include SLCTs, pleuropulmonary blastoma, renal tumors, thyroid disease including multinodular goiter, ophthalmologic and otolaryngologic tumors, central nervous system tumors, and gastrointestinal polyps [3, 5]. Although our patient had no personal or family history suggestive of cancer predisposition, her young age at diagnosis raises the possibility of an underlying DICER1‐related predisposition. Genetic counseling and DICER1 testing would therefore be relevant; however, this analysis was not available in our institution and could not be performed.

Overall, this case provides important insights into the clinical and biological spectrum of pediatric SLCTs, highlighting the unusually early age at presentation, the rare estrogen‐secreting phenotype, and the potential role of neoadjuvant chemotherapy in large tumors. It also underscores the critical importance of accurate FIGO staging in guiding therapeutic decisions, as well as the utility of inhibin B as a biomarker for disease monitoring. Given the patient's young age, the long‐term impact of treatment on her future fertility cannot yet be determined, warranting continued monitoring of ovarian function and reproductive health. These findings reinforce the need for a comprehensive, multidisciplinary approach integrating clinical, radiological, and hormonal parameters in the management and follow‐up of pediatric SCSTs.

## Author Contributions

**Sanaa Bouramdane:** conceptualization, visualization, writing – original draft, writing – review and editing, investigation, data curation. **Kenza Elmkaddem:** investigation, writing – review and editing, validation. **Nawal Hammas:** investigation, validation, writing – review and editing. **Meryem Boubbou:** investigation, validation, writing – review and editing. **Othmane Alaoui:** investigation, writing – review and editing. **Khalid Khattala:** investigation, writing – review and editing. **Youssef Bouabdellah:** investigation, writing – review and editing. **Sarra Benmiloud:** conceptualization, validation, writing – review and editing, supervision.

## Funding

The authors have nothing to report.

## Consent

Written informed consent for publication of this case report and accompanying images was obtained from the patient's parents.

## Conflicts of Interest

The authors declare no conflicts of interest.

## Data Availability

The data that support the findings of this study are available from the corresponding author upon reasonable request.
